# Identification of *PLOD3* and *LRRN3* as potential biomarkers for Parkinson’s disease based on integrative analysis

**DOI:** 10.1038/s41531-023-00527-8

**Published:** 2023-05-31

**Authors:** Xing Guo, Wenjun Hu, Zijie Gao, Yang Fan, Qianqian Wu, Weiguo Li

**Affiliations:** 1 Department of Neurosurgery, Qilu Hospital, Cheeloo College of Medicine and Institute of Brain and Brain-Inspired Science, Shandong University, 250012 Jinan, Shandong China; 2 Shandong Key Laboratory of Brain Function Remodeling, 250012 Jinan, Shandong China; 3 Department of General Practice, Central Hospital Affiliated to Shandong First Medical university, 250000 Jinan, Shandong China

**Keywords:** Parkinson's disease, Genetic testing

## Abstract

Parkinson’s disease (PD) is one of the most prevalent movement disorders and its diagnosis relies heavily on the typical clinical manifestations in the late stages. This study aims to screen and identify biomarkers of PD for earlier intervention. We performed a differential analysis of postmortem brain transcriptome studies. Weighted Gene Co-expression Network Analysis (WGCNA) was used to identify biomarkers related to Braak stage. We found 58 genes with significantly different expression in both PD brain tissue and blood samples. PD gene signature and risk score model consisting of nine genes were constructed using least absolute shrinkage and selection operator regression (LASSO) and logistic regression. *PLOD3* and *LRRN3* in gene signature were identified to serve as key genes as well as potential risk factors in PD. Gene function enrichment analysis and evaluation of immune cell infiltration revealed that *PLOD3* was implicated in suppression of cellular metabolic function and inflammatory cell infiltration, whereas *LRRN3* exhibited an inverse trend. The cellular subpopulation expression of the *PLOD3* and *LRRN3* has significant distributional variability. The expression of *PLOD3* was more enriched in inflammatory cell subpopulations, such as microglia, whereas *LRRN3* was more enriched in neurons and oligodendrocyte progenitor cells clusters (OPC). Additionally, the expression of *PLOD3* and *LRRN3* in Qilu cohort was verified to be consistent with previous results. Collectively, we screened and identified the functions of *PLOD3* and *LRRN3* based the integrated study. The combined detection of *PLOD3* and *LRRN3* expression in blood samples can improve the early detection of PD.

## Introduction

Parkinson’s disease (PD) is a common progressive neurodegenerative disorder, characterized clinically by bradykinesia, rigidity, tremor and posture instability, which seriously affecting patients’ quality of life^1^. With the aging of society, the population of PD is increasing rapidly and the social burden is aggravating. So far, the treatment strategy and overall management of PD are improved, including medication, rehabilitation therapy, exercise and surgery^2^. Previous studies indicated that earlier diagnosis and intervention may slowing the course of PD progression^3^. Although clinical diagnosis of PD is well defined depending on core manifestations and course of disease, there is still no objective biomarkers to assist detect the earliest phases of PD, especially for sporadic PD with atypical symptoms^4^. Therefore, identification and validation of reliable biomarkers for assistant diagnosis are urgently needed and important in the management and earlier intervention of PD.

Recently, comprehensive transcriptomics analysis based on blood and postmortem substantia nigra (SN) samples were performed respectively to identify potential biomarker or regulator of PD^5^. For postmortem SN transcriptome analysis, Qian Wang et al. employed a multiscale network biology approach and identified STMN2 as a key regulator of PD pathogenic pathways^6^. For blood sample transcriptome analysis, differentially gene expression between PD and normal control were screened and two optimal gene biomarker panels (CS, PRKCD, RHOG, VAMP2 and GPX3, *LRRN3*, POLR1D) were identified as potential predictor to diagnosis PD^7,8^. Besides, Marcelo et al. performed gene meta-analysis of blood samples and identified a gene-set by classification algorithms to accurately predict idiopathic PD^9^. As PD is a progressive disease, to some extent, the biomarkers improved earlier and accurate diagnosis of PD combined with clinical symptoms. However, the specificity of the biomarkers based on blood sample are poor, because it is influenced by the general condition. In addition, the biomarkers based on SN samples lack sensitivity, because SN tissue cannot be obtained from clinic and the blood sample hardly reflect the degeneration of SN.

In this study, we performed an integrative analysis of tissue and blood sample biopsies in order to screen and identify biomarkers which can balance the specificity of SN tissue and sensitivity of blood samples for diagnosis of PD.

## Results

### Integrated analysis of tissue and blood samples from PD and control patients

The detection of biomarkers only from circulating body fluids such as blood is often limited in the clinical application of Parkinson’s disease (PD) diagnosis due to poor specificity^10^. The genes upregulated in substantia nigra (SN) tissue of PD patients may serve as key drivers of the disease; however, detection of pathogenic genes is not yet possible due to the inaccessibility of brain tissue samples. It has been reported that disease-related molecules such as circulating tumor DNA (ctDNA) can be detected by liquid biopsy, which greatly improves the accuracy of early diagnosis of disease^11,12^. However, the studies using pathogenic cell-free RNA present in circulating body fluids for early diagnosis of PD are scarce.

**Fig. 1:**
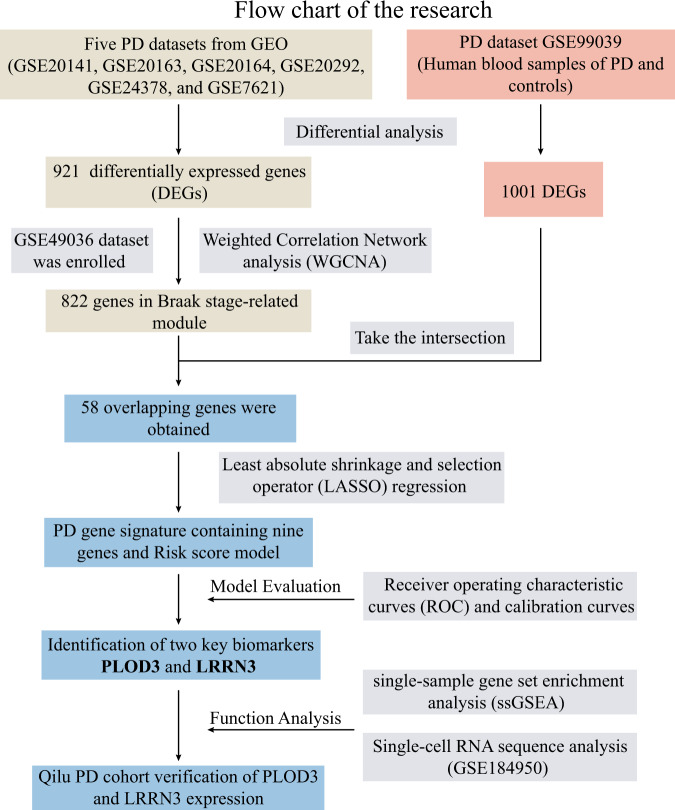
Flow chart of our research. The flow chart showing the collection of multiple data sets, dimensionality reduction, gene signature model construction and evaluation, and validation of key biomarkers.

**Fig. 2:**
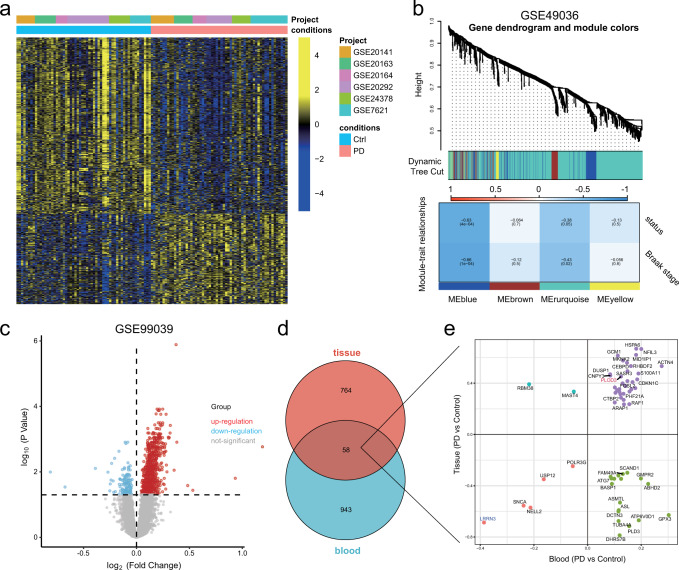
Integrated analysis of tissue and blood samples from PD and control patients. **a** Heat map visualization of 921 differentially expressed genes (DEGs) between PD and control nigrostriatal (SN) RNA. **b** The weighted gene co-expression network analysis (WGCNA) was performed with the DEGs expression data to identify the Braak stage related models. **c** Volcano map visualization of 1001 DEGs between PD and control blood samples of GSE99039. **d**, **e** Venn diagrams and scatter plots show the distribution of the overlapping 58 genes.

To simultaneously improve the sensitivity and specificity of biomarkers for the diagnosis of PD, we used an integrated analysis based on biopsy of tissue and blood samples data. The overall research workflow is illustrated in Fig. 1. Firstly, we screened the Gene Expression Omnibus (GEO, https://www.ncbi.nlm.nih.gov/geo/) database and six independent studies were identified, which contained RNA sequence data from human substantia nigra (SN). We then integrated data from six studies using the “ComBat” algorithm to correct for non-biotechnical biases that contribute to batch effects. Next, we performed differential analysis between the PD and control group (PD: 59; Control: 58), and obtained 921 differentially expressed genes (DEGs) (Fig. 2a). The Braak staging system represented the spread of Lewy pathology^13^, which is corelated with evolution of clinical symptoms in PD patients, such as bradykinesia, rigidity, cognitive decline etc^14^. We further introduced GSE49036 which contains information on the Braak stage for 28 samples. Weighted Correlation Network analysis (WGCNA) was performed with the obtained 921 DEGs. Blue and turquoise module (822 genes) that significantly correlated with Braak stage were identified as the “Braak stage-related module in PD” (Fig. 2b). Additionally, a blood-based gene expression profiling study (GSE99039) was enrolled in our research. We identified 1,001 DEGs between blood samples from PD and controls (Fig. 2c). Then we took the intersection of the 1001 DEGs and “Braak stage-related module” and finally obtained overlapping 58 genes for further analysis (Fig. 2d, e).

### Identification of a PD gene signature consisting 9 biomarker genes

**Fig. 3:**
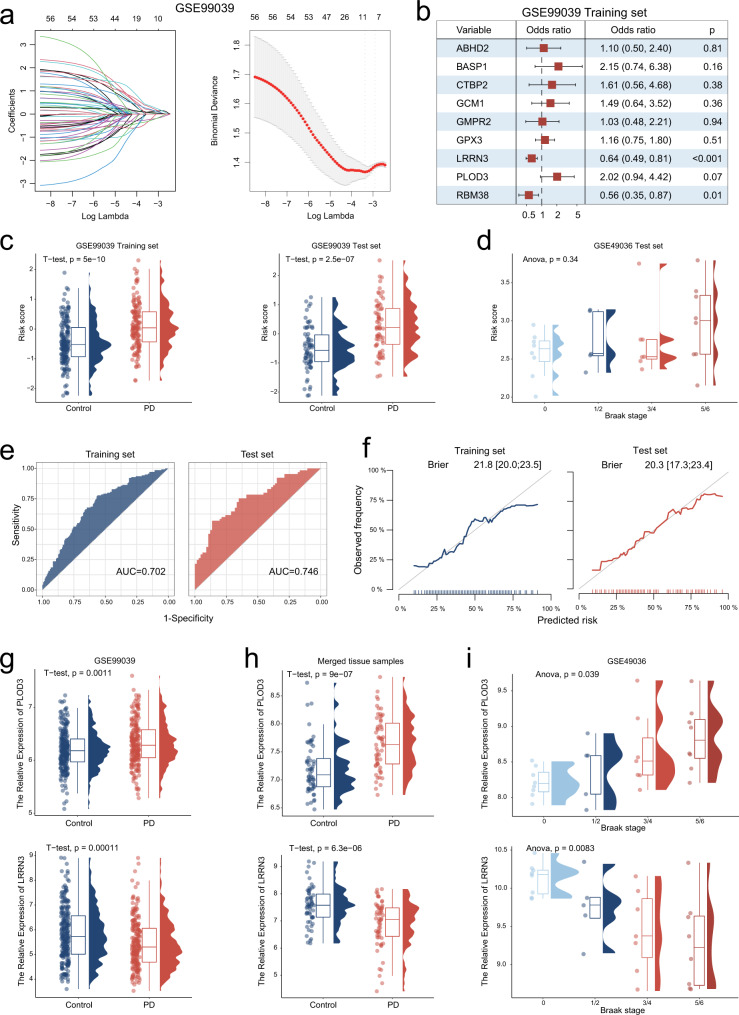
Identification of a PD gene signature consisting 9 biomarker genes. **a** Least absolute shrinkage and selection operator (LASSO) coefficient profiles (*y*-axis) of the 58 overlapping genes (left panel). The dashed line on the left represents the optimal value of λ, which corresponds to the number of genes on the x-axis (right panel). **b** Multivariate logistic regression model analysis, which included the nine genes in the training set of GSE99039. The forest plot displays the odds ratio (OR) values and their 95% confidence intervals from various genes. Each square represents a gene, with the position of the square indicating the OR value and the horizontal line representing the 95% confidence interval. **c** The differences of risk score between PD and control groups for training set and test set of GSE99039. **d** The differences of risk score between different braak stage PD patients of GSE49036 **e** Receiver operating characteristic (ROC) curves for the risk score model both in the training and test sets. **f** The calibration curves of risk score predictions for the training and test sets are close to the ideal performance (45 degrees line). **g**, **h** The differences of *PLOD3* and *LRRN3* expression between PD and control groups of GSE99039 (**g**) and merged SN tissue samples (**h**), respectively. **i** The differences of *PLOD3* and *LRRN3* expression between different Braak stages groups of GSE49036. Boxplots summarize the distribution of the data. The box represents the interquartile range, with the horizontal line inside the box indicating the median. The whiskers extend to the minimum and maximum values within 90% of the data range and the shape of the violin provides insights into the data distribution.

To determine the key biomarkers in PD blood, we further performed least absolute shrinkage and selection operator (LASSO) regression algorithm for dimensionality reduction. The 438 participants of GSE99039 were randomly divided into two parts (7:3) for the training set and test set, respectively. The 58 genes were entered into LASSO regression analysis and 9 genes (*ABHD2*, *BASP1*, *CTBP2*, *GCM1*, *GMPR2*, *GPX3*, *LRRN3*, *PLOD3*, and *RBM38*) were finally selected using an optimal λ value (λ.1se = 0.0498) (Fig. 3a). We further performed multivariate logistic regression analysis and constructed a risk score model using the “predict” function based on the nine genes for training and test sets (Fig. 3b and Supplementary Figure 1). The risk scores of PD patients were significantly higher than those of control patients in both the training and test sets (Fig. 3c). In addition, we further compared the risk scores of 28 PD patients with different Braak stages in the GSE49036 dataset. Although patients with PD in Braak stage 5/6 appeared to be higher than the other groups, the statistical results were not significant due to the limitations of the sample size (Fig. 3d). ROC analysis demonstrated that the predictive capacity of risk score was powerful, whereas, the AUC values were 0.702 and 0.746 in training and test sets, respectively (Fig. 3e). Calibration curves of risk score in training set and test set were close to the ideal performance (45-degree gray line), which indicated the predictive stability of risk score (Fig. 3f). Based on the expression of nine genes in GSE99039 and merged tissue samples, we performed correlation analysis and visualized using network plot (Supplementary Figure 1). Collectively, a PD gene signature consisting nine genes was identified and the risk score model could predict the risk of PD incidence with a higher degree of accuracy.

### Identification of two key biomarkers *PLOD3* and *LRRN3*

When in comparison of the nine key genes expression between different Braak stages groups, we observed that only *PLOD3* and *LRRN3* were significantly associated with Braak stages (Supplementary Figure 2 and Fig. 3i). Additionally, the expression differences of *PLOD3* and *LRRN3* between PD and control groups were consistent in both blood and SN tissue samples (Supplementary Fig. 2 and Fig. 3g, h). That is, *PLOD3* expression was up-regulated not only in blood samples but also in SN tissues of PD patients, whereas the expression of *LRRN3* was down-regulated in both blood and SN tissue samples. *PLOD3* and *LRRN3* may act as PD-driven and PD-suppressed molecules, respectively, which are also important biomarkers detected in blood samples. The protein encoded by *PLOD3* is a membrane-bound enzyme that is localized to the cisternae of the rough endoplasmic reticulum. *PLOD3* has been widely studied in gastric cancer^15^, non-small cell lung cancer^16^ and triple-negative breast cancer^17^. In addition, a recent study reported that *PLOD3* is associated with immune cell infiltration in colorectal cancer^18^. However, studies on the biomarker significance and pathogenic mechanisms of *PLOD3* in PD are not available. *LRRN3* was identified as a potential diagnostic biomarker for PD patients’ blood samples^8^. As CSF is a biological fluid that surrounds the brain and spinal cord and can provide valuable information about the nervous system’s functioning, we further utilized GSE141578 data to analyze biomarkers in CSF. The results showed that *PLOD3* and *LRRN3* were expressed relatively low in CSF, and these expressions were not significantly different between control and PD groups (Supplementary Fig. 2). However, more exploration needs to be taken to investigate the role of *PLOD3* and *LRRN3* in CSF for PD diagnosis. Our results indicated that *LRRN3* expression was downregulated in both blood and SN tissue samples from PD patients. Taken together, the above results suggest that *PLOD3* and *LRRN3* are not only implicated in the diagnostic significance of PD, but also may be involved in the physiological mechanisms of PD pathogenesis.

### Correlation analysis of *PLOD3* and *LRRN3* with immune cell infiltration in PD

Further analysis was performed to define the function of *PLOD3* and *LRRN3* in PD. As inflammation and immune microenvironment were reported to play a determinant role in the pathogenesis of PD, we focused on the difference of immune state between PD and control. To evaluate the immune status of SN tissue, we calculated the immune score, stromal score and estimate score using “estimate” package. PD patients exhibited higher immune scores and estimated scores compared to control patients, and the estimated scores were positively correlated with Braak stages (Supplementary Fig. 3). We further performed ssGSEA to evaluate the immune cells infiltration. The results demonstrated that pDCs, T helper cells, TIL etc. were higher infiltrated in SN tissue of PD patients than control. The infiltration of B cells, neutrophils, TIL, and Treg were positively correlated with Braak stages, while CD8 T cells were negatively correlated (Supplementary Fig. 3).

Based on the median values of *PLOD3* and *LRRN3* expression, we stratified the PD patients into high and low groups. PD patients with higher *PLOD3* expression exhibited higher stromal score, immune score and estimate score, whereas PD patients with higher *LRRN3* expression exhibited the opposite immune score profile (Supplementary Fig. 4). Additionally, PD patients with higher *PLOD3* expression showed significantly higher infiltration of pDCs, T helper cells, TIL, macrophages, while PD patients with higher *LRRN3* expression showed the opposite immune cell infiltration profile (Supplementary Fig. 4). Totally, our results demonstrated that *PLOD3* was implicated in immune response activation status and higher inflammatory cell infiltration, whereas *LRRN3* was implicated in relative immune response suppression status.

### The expression of *PLOD3* and *LRRN3* exhibited differential distribution of cell subpopulations

**Fig. 4:**
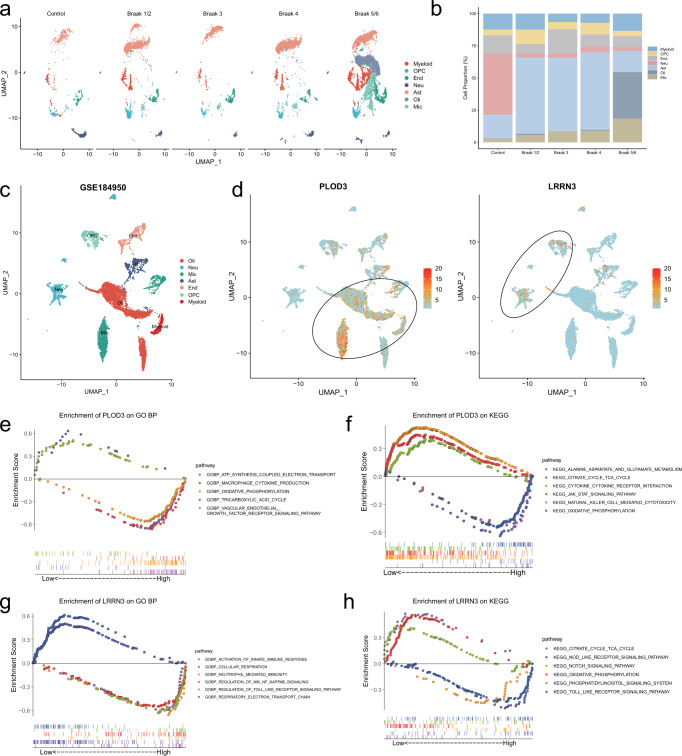
Single-cell RNA analysis reveals the functions of *PLOD3* and *LRRN3*. **a** UMAPs of control and different Braak stages PD brain samples. **b** Cluster composition of myeloid, oligodendrocyte progenitor cells (OPC), endothelial cells (End), neurons (Neu), astrocytes (Ast), oligodendrocytes (Oli), and microglia (Mic) from snRNA sequencing. **c**, **d** Single-cell RNA sequencing of GSE184950 visualizing UMAP cell clusters, *PLOD3* and *LRRN3* expression. The cellular subpopulation expression of the *PLOD3* and *LRRN3* has significant distributional variability. The expression of *PLOD3* was more enriched in astrocytes, microglia and oligodendrocytes cell clusters, whereas *LRRN3* was more enriched in neurons and oligodendrocyte progenitor cells clusters. **e**–**h** The combined GSEA curves using GO-BP and KEGG analysis visualize the functions of *PLOD3* and *LRRN3*.

We leveraged a single-cell nuclear transcriptomics data (GSE184950) of human SN to analyze the cell subpopulation expression distribution of *PLOD3* and *LRRN3*. We determined and annotated the cell types of these cell clusters by examining the expression of known gene markers of brain cell type. A total of seven cell types were identified, which consisted oligodendrocytes (Oli), neurons (Neu), microglia (Mic), astrocytes (Ast), endothelial cells (End), oligodendrocyte progenitor cells (OPC), and myeloid. As the Braak stages increased, the proportion of microglia and oligodendrocytes significantly increased, while the proportion of neuronal cells decreased significantly compared to the control group (Fig. 4a, b). We further examined the expression distribution of *PLOD3* and *LRRN3*. *PLOD3* expression was more abundant in astrocytes, microglia and oligodendrocyte populations, whereas *LRRN3* was more enriched in neuronal and oligodendrocyte progenitor cell populations (Fig. 4c, d). The above results consisted with our previous results that *PLOD3* was implicated in higher inflammatory cell infiltration, while *LRRN3* was implicated in relative immune suppression status. The GSEA analysis also demonstrated that *PLOD3* was negative correlated with cellular metabolism-related functions such as tricarboxylic acid cycle and oxidative phosphorylation (Fig. 4e, f), whereas *LRRN3* was positively correlated with cellular metabolic functions such as cellular respiration and respiratory electron transport chain (Fig. 4g, h). We concluded that *PLOD3* was implicated in suppression of cellular metabolic function and inflammatory cell infiltration, whereas *LRRN3* exhibited opposite effects.

### Validation the expression of *PLOD3* an *LRRN3* in blood samples

**Fig. 5:**
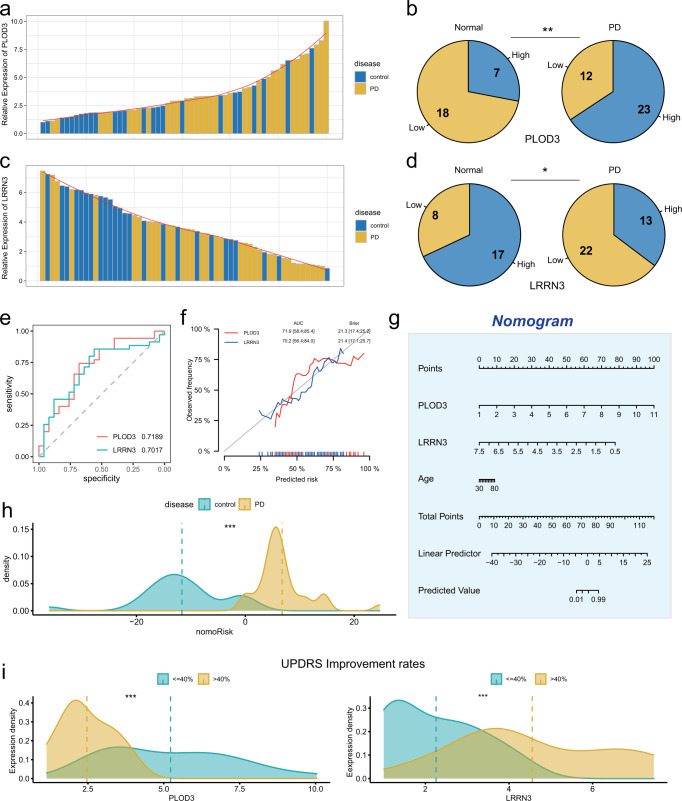
Validation the expression of *PLOD3* an *LRRN3* in blood samples. **a**, **c** The bar chart showing the relative expression of *PLOD3* and *LRRN3* in PD and control blood samples. **b**, **d** Pie charts showing the distribution of *PLOD3*-Low/High (**b**) and *LRRN3*-Low/High (**d**) expression in control and PD blood samples. **e**, **f** ROC curves and calibration curves for the expression of *PLOD3* and *LRRN3* in Qilu cohort. **g** Nomogram based on the expression of *PLOD3*, *LRRN3* and age for predicting the incidence of PD in Qilu cohort. **h** The differences of nomoRisk between control and PD. **i** The differences in *PLOD3* and *LRRN3* expression between PD patients with high and low UPDRS improvement rates.

To further validate the diagnostic efficacy of *PLOD3* and *LRRN3* for PD, we collected blood samples derived from 35 patients with a clinical diagnosis of PD and 23 controls. qRT-PCR was performed to examine the relative expression of *PLOD3* and *LRRN3*. As the results demonstrated, in PD and control cohorts, a greatly increased level of *PLOD3* was observed to be more common in PD blood samples than in control (Fig. 5a, b). In contrast, samples with higher *LRRN3* expression were more numerous in the control group than in the PD group (Fig. 5c, d). Then the ROC analysis was performed to assess the predictive accuracy of *PLOD3* and *LRRN3*, with AUC values of 0.7189 and 0.7017 for *PLOD3* and *LRRN3*, respectively. (Fig. 5e). The calibration curves of *PLOD3* and *LRRN3* were close to the ideal performance, which indicated a powerful and robust predictive capacity of *PLOD3* and *LRRN3* for PD (Fig. 5f). We constructed a nomogram model by integrating *PLOD3* and *LRRN3* expression together to further improve the diagnostic efficacy of PD (Fig. 5g). Density plot was used to visualize the nomoRisk score and PD patients exhibited a significantly higher nomoRisk score than control (Fig. 5h). Unified Parkinson’s Disease Rating Scale (UPDRS) score is widely used to assess cognitive function, motor function and linguistic function of PD patients. In addition, UPDRS improvement rates after dopamine challenge test can be used to assess the sensitivity to anti-PD drugs and outcomes for Deep Brain Electrical Stimulation (DBS) surgery for PD patients.

Our results showed that PD patients with higher UPDRS improvement rates after dopamine challenge test had lower *PLOD3* and higher *LRRN3* in their blood samples, which further reveals the significance of examining *PLOD3* and *LRRN3* (Fig. 5i). Collectively, we enrolled Qilu cohort to validate the expression of *PLOD3* and *LRRN3* in blood samples, which exhibited excellent predictive efficiency.

## Discussion

As a progressive neurodegenerative disorder, clinical symptoms of PD appearance lag behind the pathology onset of SN. The current diagnostic criteria of PD relying on clinical symptoms go against the detection and intervention of early stage. In order to screen and identify biomarkers of PD for early intervention and slowing disease progression, we performed a differential analysis based on six merged cohorts of SN transcriptome and a single-cell atlas of SN transcriptome, combined with blood sample transcriptome. In addition, WGCNA was used to identify biomarkers related to Braak stage of PD. LASSO and logistic regression were used to construct a novel 9 genes-set signature. Two key biomarkers *PLOD3* and *LRRN3* were identified as potential predictors to diagnosis PD. Finally, *PLOD3* and *LRRN3* were verified in our hospital PD cohort of blood samples and may be related to clinical sensitivity to anti-PD drugs.

Over the past decade, multiple studies focused on diagnostic biomarkers of PD and proved the potential cerebrospinal fluid (CSF) and blood biomarkers could reflect the pathophysiology of PD^19^. Species of α-synuclein as central regulation molecules in the pathophysiological mechanisms were commonly measured differential expression in CSF and blood of PD compared with control. The diagnostic accuracy of α-synuclein in the blood was disturbed by red blood cell contamination, which caused inconsistence results of serum and plasma from different scholars. Although the measurement of α-synuclein in CSF exclude the red blood cell contamination, unsatisfactory diagnostic accuracy of α-synuclein in CSF for PD patient was obtained. Therefore, reliable blood biomarker as a non-invasive tool still be necessary for early diagnosis of PD in clinic. Biomarkers are measurable indicators of biological processes or disease states. In the future, the use of biomarkers in blood and cerebrospinal fluid or a combination of both may become more widespread and refined. Advances in technology and analytical methods will allow for the identification of new biomarkers and improved sensitivity and specificity of existing biomarkers. Biomarkers may also be used to identify subtypes of diseases, allowing for personalized treatment strategies. Additionally, biomarkers may be used to monitor treatment response, allowing for early identification of treatment effectiveness or the need for alternative therapies. In this study, on the one hand, we integrated the transcriptome of SN to screen accurate and stable biomarkers which correlated with the progressive pathology of Braak stage. On the other hand, we chose the co-differential expressed biomarkers in blood and SN, which may accurately reflect the pathophysiology of PD. Based on the co-differential expressed biomarkers, a PD gene signature consisting 9 key genes was identified, including *LRRN3* and *PLOD3*. The risk score of the gene signature could predict the risk of PD incidence with a higher degree of accuracy.

Leucine-rich repeat neuronal protein 3 (*LRRN3*) enriched in the cerebral cortex participated in regulating the synaptic connection^20^. The expression of *LRRN3* were lower in PD compared with control and reported to decline with age^21^, indicating progression of neurodegeneration may influence *LRRN3* expression. Recently, whole-blood transcriptome analysis revealed that *LRRN3* was top smoking-related highly expressed genes in smokers. More interestingly, plenty clinical studies showed that smoking took a causally protective effect on the risk of PD^22,23^, indicating the potential etiology role of *LRRN3* in PD. Procollagen-lysine, 2-oxoglutarate 5-dioxygenase 3 (*PLOD3*), one of the lysyl hydroxylases family, involved in catalyze the formation of collagen cross-link and deposition^24^. Overexpression of *PLOD3* were detected in many human diseases, including collagen-related diseases and cancers. Recent research reported that the extracellular matrix consisted more collagen in prefrontal cortex of PD compared with control. The increasing *PLOD3* expression may resulting in the enrichment of collagen of SN of PD, and associated with the pathogenesis of PD. In this study, the potential biomarkers of *LRRN3* and *PLOD3* were respectively proved to be highly and lowly expressed in blood consistence with SN of PD. Furthermore, we confirmed the diagnostic efficacy of *PLOD3* and *LRRN3* for PD via collected blood samples derived from 35 patients with a clinical diagnosis of PD and 23 controls in our hospital. Moreover, the expression of *PLOD3* and *LRRN3* correlated with the UPDRS improvement rates after dopamine challenge test, which indicated the potential prognostic value for PD patients. However, the detail function of *LRRN3* and *PLOD3* in PD progression is not elucidated clearly.

Dysregulation of the immune microenvironment plays a determinant role in the pathogenesis of Parkinson’s disease. Neuroinflammation has been treated as a hallmark of PD and plays a critical role in PD pathogenesis through triggering neuronal dysfunction and death^25^. Specifically, microglia can be activated and further migrate to the brain through a compromised BBB, and contribute to disease progression by mediating the immune pathways and interacting with α-synuclein. Therefore, the innate immune responses triggered by microglia can cause neuronal death and disease progression. Our further analysis showed that PD exhibited activation status of immune response and higher immune cell infiltration. Besides, our results demonstrated that *PLOD3* was implicated in immune response activation status, whereas *LRRN3* was implicated in immune response suppression status. More importantly, according to single-cell transcriptome analysis, *PLOD3* was enriched in microglia cells of PD contrast to control, indicating the potential role of *PLOD3* in immune response regulated by microglia during the pathological progression of PD. Vice versa, *LRRN3* was mainly expressed in neurons, hinting the decreasing expression of *LRRN3* correlated with the infiltration of immune cells was unfavorable for the protection of neurons. Meanwhile, the latest bioinformatic studies showed that *PLOD3* and *LRRN3* composed the gene signature of immune cell infiltration^26,27^. As the potential regulation role during the progressive pathology of PD, the *PLOD3* and *LRRN3* may have the early diagnosis value for PD.

In conclusion, our study constructed an accuracy 9-genes PD signature model to predicted the risk of PD. Among the 9 genes, *PLOD3* and *LRRN3* were identified and validated to be reliable biomarkers for diagnosis of PD in blood samples. According to the functional enrichment analysis, *PLOD3* and *LRRN3* correlated with immune infiltration of SN in PD. In the future, the potential role and mechanism of *PLOD3* and *LRRN3* in regulating immune infiltration during the progression of PD could be focused on research.

## Methods

### Data collection and processing

For human substantia nigra (SN) RNA sequencing profiles, a total of six independent GEO datasets (GSE20141, GSE20163, GSE20164, GSE20292, GSE24378, and GSE7621) were enrolled to this study and the “ComBat” algorithm was used to correct for non-biotechnical biases that contribute to batch effects. GSE49036 was used to identify biomarkers related to Braak stage based. For human blood samples of PD and controls, GSE99039 was enrolled. For single-cell RNA sequencing analysis, GSE184950 was enrolled.

For qRT-PCR of blood samples in Qilu cohort, 35 patients with a clinical diagnosis of PD and 23 controls with no history of major brain illness from June 2021 to December 2022 in Department of Functional Neurosurgery were included.

### Differential analysis

The “limma” R package was leveraged to identify differential expressed genes (DEGs) between PD and control groups. The significance criterion for DEGs was *p*-value < 0.01.

### Weighted Correlation Network analysis (WGCNA)

WGCNA was used to construct a scale-free co-expression network using the R package ‘WGCNA’ and to identify a gene module that is mostly correlated with Braak stage. The obtained DEGs between PD and control groups were involved in WGCNA to identify “Braak stage-related module” in PD.

### Immune status and immune cell infiltration analysis

ESTIMATE was used to calculate the immune score, stromal score and estimate score according to the R package “Estimate”. single-sample gene set enrichment analysis (ssGSEA) was used to identify tumor immune-infiltrating cell abundance in SN tissues of PD patients. The markers of 28 immune-related cells and types were obtained from the dataset of Bindea et al.^28^.

### Gene functional annotation based on gene set enrichment analysis

To analyze the differences in biological processes of *PLOD3* and *LRRN3*, gene set enrichment analysis (GSEA) enrichment was performed via the “GSEA_4.1.0” software. We downloaded the gene set “c5.go.bp.v7.4” as well as “c2.cp.kegg.v7.4” from the Molecular Signatures Database (MSigDB, https://www.gsea-msigdb.org/gsea/msigdb/index.jsp) for GSEA.

### Single Cell nuclear RNA sequencing analysis

The single-cell RNA-sequencing of gliomas were downloaded from Gene Expression Omnibus (GEO, https://www.ncbi.nlm.nih.gov/geo/, GSE184950) and analyzed using R package “Seurat 4.1.0”. Method “UMAP” was applied for the visualization of different cell clusters.

### Real-time quantitative RT-PCR (qRT-PCR)

The total RNA was extracted by TRIzol (Invitrogen, USA) following manufacturer’s protocol. We performed reverse transcription using high-capacity-cDNA Reverse Transcription Kit (Toyobo, China) according to the protocol. The PCR primer pairs’ sequences were: 5′-CAGAGATGGAGCACTACGGC-3′ (forward) and 5′-CTTGGTGTGGTAACCGGGAA-3′ (reverse) for *PLOD3*; 5′-CCCATCAGGTGTGACTGTGT-3′ (forward) and 5′-GCCGAACATTCTGACCTTGG-3′ (reverse) for *LRRN3*. Mx-3000P Quantitative PCR System (Applied Biosystems, USA) was leveraged for qRT-PCR.

### Statistical analysis

We leveraged R 4.1.3 to perform all statistical analysis. We performed Pearson correlation algorithm to evaluate the correlation between different groups. The normality of distribution and the homogeneity of variance were proved by Shapiro–Wilk normality test and Bartlett test, respectively. Student’s *t* test and one-way ANOVA were performed to compare the certified data (significance > 0.1) between two groups and more than two groups, respectively. *P*-value < 0.05 were accepted as statistically significant (*p-value < 0.05; **p-value < 0.01; ***p-value < 0.001).

## Supplementary information

Supplementary Material

## Data Availability

All of the publicly available data used in this study are listed in Supplementary Table 1. Written requests for access to the Qilu Parkinson’s cohort data reported in this paper will be considered upon request to the corresponding author GLW and first author XG, as long as the appropriateness of data use is determined. If deemed appropriate, a data sharing agreement will be signed prior to providing a fully de-identified version of the dataset used for analysis.

## Code availability

R code used for dimensionality reduction is available from the authors upon request.
