# Supplementary material for: Identification of PLOD3 and LRRN3 as potential biomarkers for Parkinson’s disease based on integrative analysis
Source: NPJ Parkinsons Dis. 2023 May 31;9:82. doi: 10.1038/s41531-023-00527-8 (PMC10232497; doi:10.1038/s41531-023-00527-8)
Supplement: Supplementary file 1 — Supplementary Material [file 41531_2023_527_MOESM1_ESM.pdf]

Supplementary Table and Figures

Platform	GEO accession	Sample size		Contributors	Date	Identifier/ URL
		Control	PD			
GPL570	GSE99039	233	205	Amar et al.	2017	https://www.ncbi.nlm.nih.gov/geo/query/acc.cgi?acc=GSE99039
GPL570	GSE7621	9	16	Ffrench et al.	2007	https://www.ncbi.nlm.nih.gov/geo/query/acc.cgi?acc=GSE7621
GPL570	GSE20141	8	10	Middleton et al.	2010	https://www.ncbi.nlm.nih.gov/geo/query/acc.cgi?acc=GSE20141
GPL96	GSE20163	9	8	Miller et al.	2010	https://www.ncbi.nlm.nih.gov/geo/query/acc.cgi?acc=GSE20163
GPL96	GSE20164	5	6	Hauser et al.	2010	https://www.ncbi.nlm.nih.gov/geo/query/acc.cgi?acc=GSE20164
GPL96	GSE20292	18	11	Middleton et al.	2010	https://www.ncbi.nlm.nih.gov/geo/query/acc.cgi?acc=GSE20292
GPL1352	GSE24378	9	8	Cantuti et al.	2010	https://www.ncbi.nlm.nih.gov/geo/query/acc.cgi?acc=GSE24378
GPL570	GSE49036	8	20	Dijkstra et al.	2015	https://www.ncbi.nlm.nih.gov/geo/query/acc.cgi?acc=GSE49036
GPL24676	GSE184950	10	24	Wang et al.	2022	https://www.ncbi.nlm.nih.gov/geo/query/acc.cgi?acc=GSE184950

Supplementary Table 1. Description of the included GEO cohorts

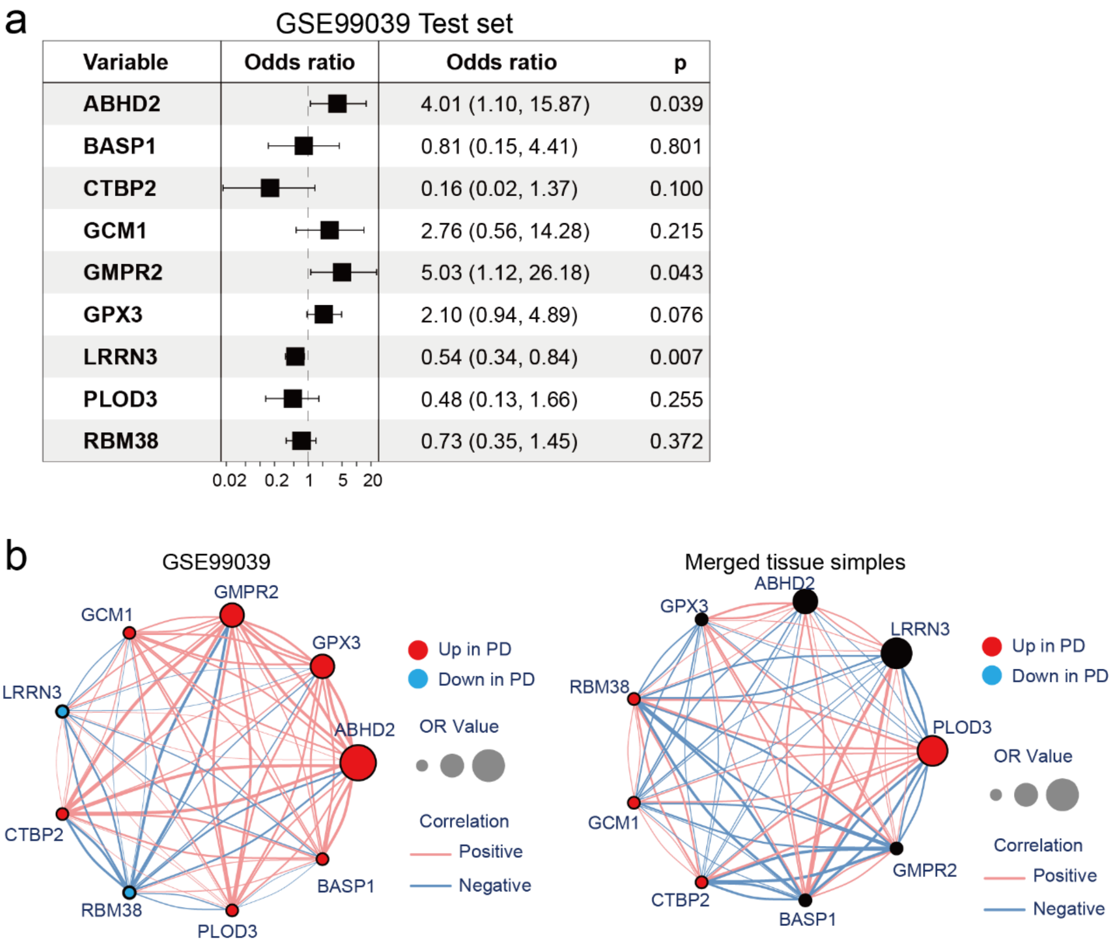

Supplementary Figure 1. a Multivariate logistic regression model analysis, which included the nine genes in the test set of GSE99039. The forest plot displays the odds ratio (OR) values and their 95% confidence intervals from various genes. Each square represents a gene, with the position of the square indicating the OR value and the horizontal line representing the 95% confidence interval. **b** Network plot visualized the correlation between nine genes from GSE99039 and merged GEO datasets, respectively.

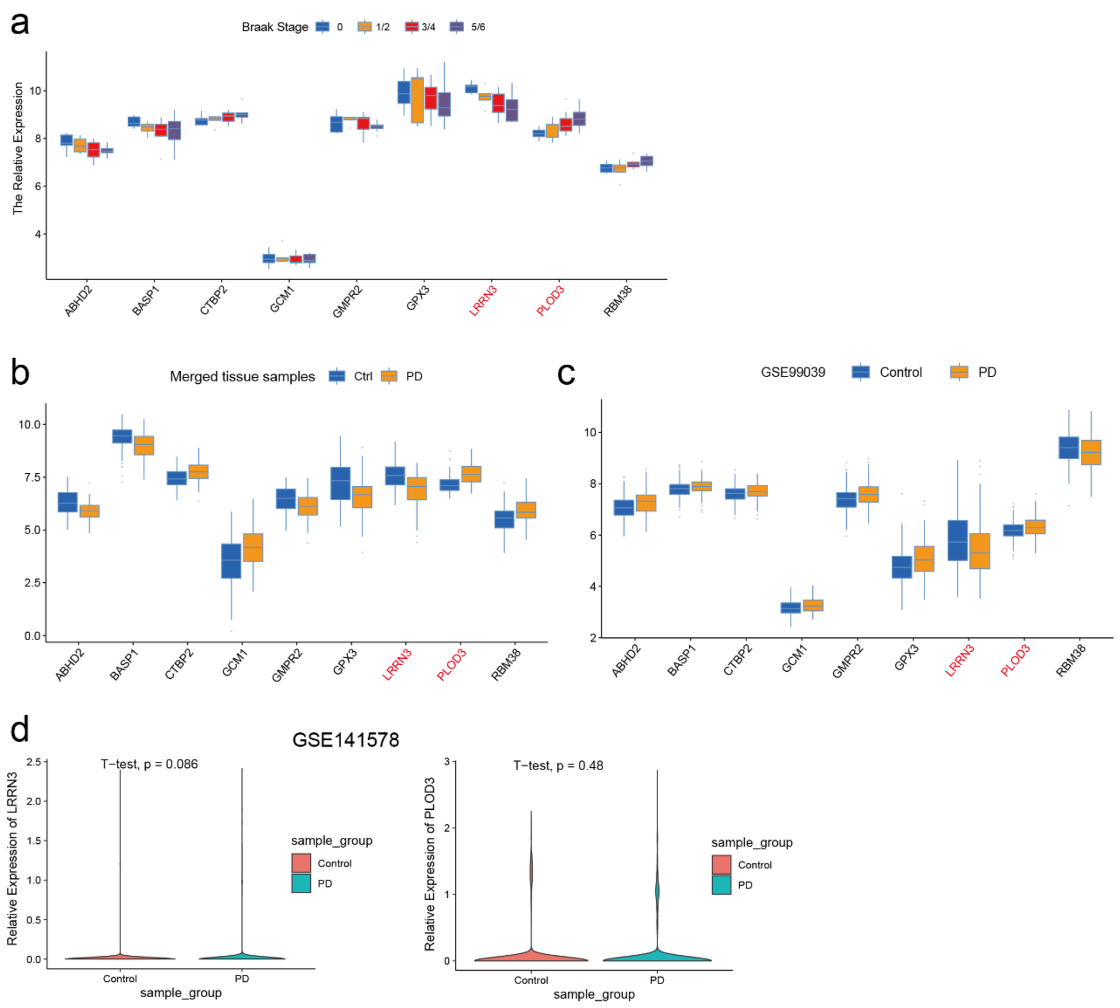

Supplementary Figure 2. a The expression difference of nine genes from different Braak stages samples. **b, c** The expression difference of nine genes between control and PD groups of merged GEO datasets (**b**) and GSE99039 (**c**), respectively. **d** The expression difference of LRRN3 and PLOD3 in the CSF between control and PD patient groups. Boxplots summarize the distribution of the data. The box represents the interquartile range, with the horizontal line inside the box indicating the median. The whiskers extend to the minimum and maximum values within 90% of the data range.

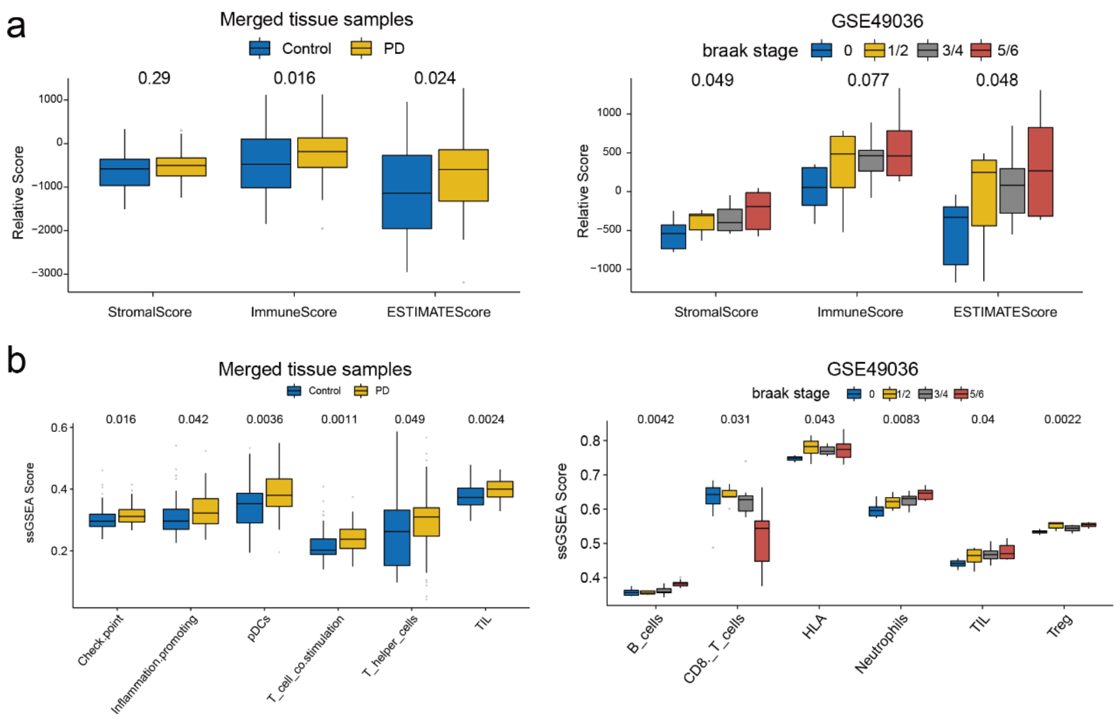

Supplementary Figure 3. a The difference of stromal score, immune score and ESTIMATE score between indicated groups. **b** The difference of immune infiltration between indicated groups of merged GEO datasets and GSE49036, respectively. The box represents the interquartile range, with the horizontal line inside the box indicating the median. The whiskers extend to the minimum and maximum values within 90% of the data range.

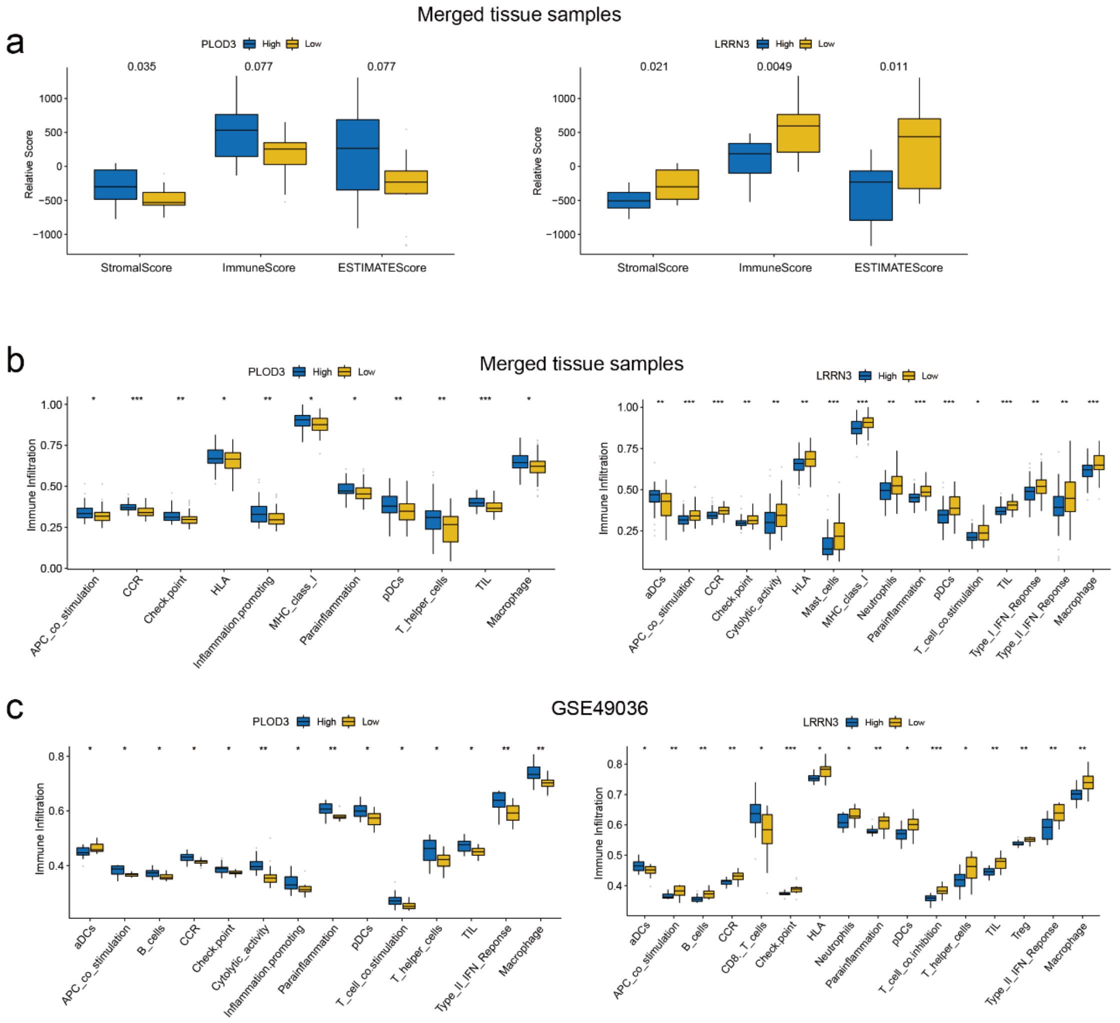

Supplementary Figure 4. a The difference of stromal score, immune score and ESTIMATE score between high and low PLOD3 (left panel) or LRRN3 (right panel) groups. **b, c** The difference of immune infiltration between high and low PLOD3 or LRRN3 groups of merged GEO datasets (**b**) and GSE49039 (**c**), respectively. The box represents the interquartile range, with the horizontal line inside the box indicating the median. The whiskers extend to the minimum and maximum values within 90% of the data range. P-value < 0.05 were accepted as statistically significant (*p-value < 0.05; **p-value < 0.01; ***p-value < 0.001).
